# Effect of simulated transport vibration on the quality of shiitake mushroom (*Lentinus edodes*) during storage

**DOI:** 10.1002/fsn3.2094

**Published:** 2020-12-28

**Authors:** Fei Tao, Wenwei Chen, Zhenbao Jia

**Affiliations:** ^1^ College of Standardization China Jiliang University Hangzhou China; ^2^ Key Laboratory of Marine Food Quality and Hazard Controlling Technology of Zhejiang Province China Jiliang University Hangzhou China

**Keywords:** foam cushion material, quality degradation, shiitake mushroom *(Lentinus edodes)*, transport vibration

## Abstract

The quality and shelf life of mushrooms are critical to their commercial viability. In this study, the effects of simulated transport vibration on postharvest quality of shiitake mushrooms (*Lentinus edodes*) were assessed over 12 days of storage. Furthermore, the protective performance of foam cushion material used in packaging during simulated transport was evaluated. Changes in respiration rate, weight loss, browning index, firmness, and malondialdehyde content were measured following vibration treatment. The results revealed that simulated transport vibration contributed to the deterioration of quality of shiitake mushrooms during storage, and the foam cushion material had a protective influence on the maintenance of shiitake mushroom quality. Taken together, our findings suggest that the foam cushion material used in packaging has the potential to improve the quality of shiitake mushrooms and extend their shelf life.

## Graphical abstract

Vibrations caused quality deterioration of shiitake mushroom. Foam cushion material delayed quality deterioration.

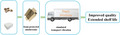

**Highlights**

- Vibrations caused quality deterioration of shiitake mushroom.
- Foam cushion material protected the color of shiitake mushroom.
- Foam cushion material delayed quality deterioration.

## 1 INTRODUCTION

Postharvest losses of fresh fruits and vegetables during distribution in China have been estimated to be as high as 30% of the initial harvested products (Zhou et al., 2007). In the past decade, considerable attention has been paid to the loss of fresh produce due to vibrations during road transportation (Fadiji et al., 2016; Fernando et al., 2020). Mechanical vibration caused by potholes and unevenness of the road during transportation is an important factor affecting the sensory quality and the marketability of produce. Tissue damage resulting from transport vibration has been widely reported in bananas (Fernando et al., 2019), apples (Luo et al., 2012), tomatoes (Wu & Wang, 2014), pears (Zhou et al., 2007), and cucumbers (Ariana et al., 2006). Additionally, several studies revealed that vibrations during transportation cause changes in chemical composition and physiological reactions of fruits after harvest (Ahmadi et al., 2010; Zhou et al., 2007). Cushion material is gaining increasing attention due to the ability to attenuate transmission of vibration. Packaging with cushion materials, such as foam net and paper wrap, has been a commercially available approach for extending shelf life and reducing tissue damage during road transportation, because such packaging can protect fresh products against mechanical damage (Jarimopas et al., 2007; Lin et al., 2020).

Mushrooms are widely consumed throughout the world and are considered an important source of dietary fiber, natural antioxidants, and vitamins. Because mushrooms have no cuticle layer to provide protection from mechanical damage, water loss, or microbial attack, they are one of the most perishable food products, with a shelf life of 3–4 days at ambient temperature (Fernandes et al., 2012; Wrona et al., 2015). In Asian countries, shiitake mushroom (*Lentinus edodes*) is one of the most economically important varieties of edible mushrooms owing to its unique flavor and texture. Furthermore, shiitake mushrooms have many beneficial effects on human health, including antitumor (Ya, 2017), antiaging (Xu et al., 2017), and antioxidant effects (Zhu et al., 2018). Expansion of the supply chain due to market globalization escalates the risk of prolonged exposure of fresh shiitake mushrooms to vibrations occurring during transport. Similar to other fruits and vegetables, it is desirable to extend the shelf life of shiitake mushrooms. However, the exact influence of vibrations on shiitake mushroom quality during storage needs to be understood. Foam cushioning materials are low cost, lightweight, and energy absorbent materials used for food packaging. However, limited information is available on the protective effects of such foam cushioning material on the quality of shiitake mushrooms.

Therefore, the objective of this study was to investigate the effects of simulated transport vibrations on the quality of shiitake mushrooms during storage. Furthermore, the protective effect of foam cushioning material was also evaluated.

## 2 MATERIALS AND METHODS

### 2.1 Mushrooms and vibration

**FIGURE 1:**
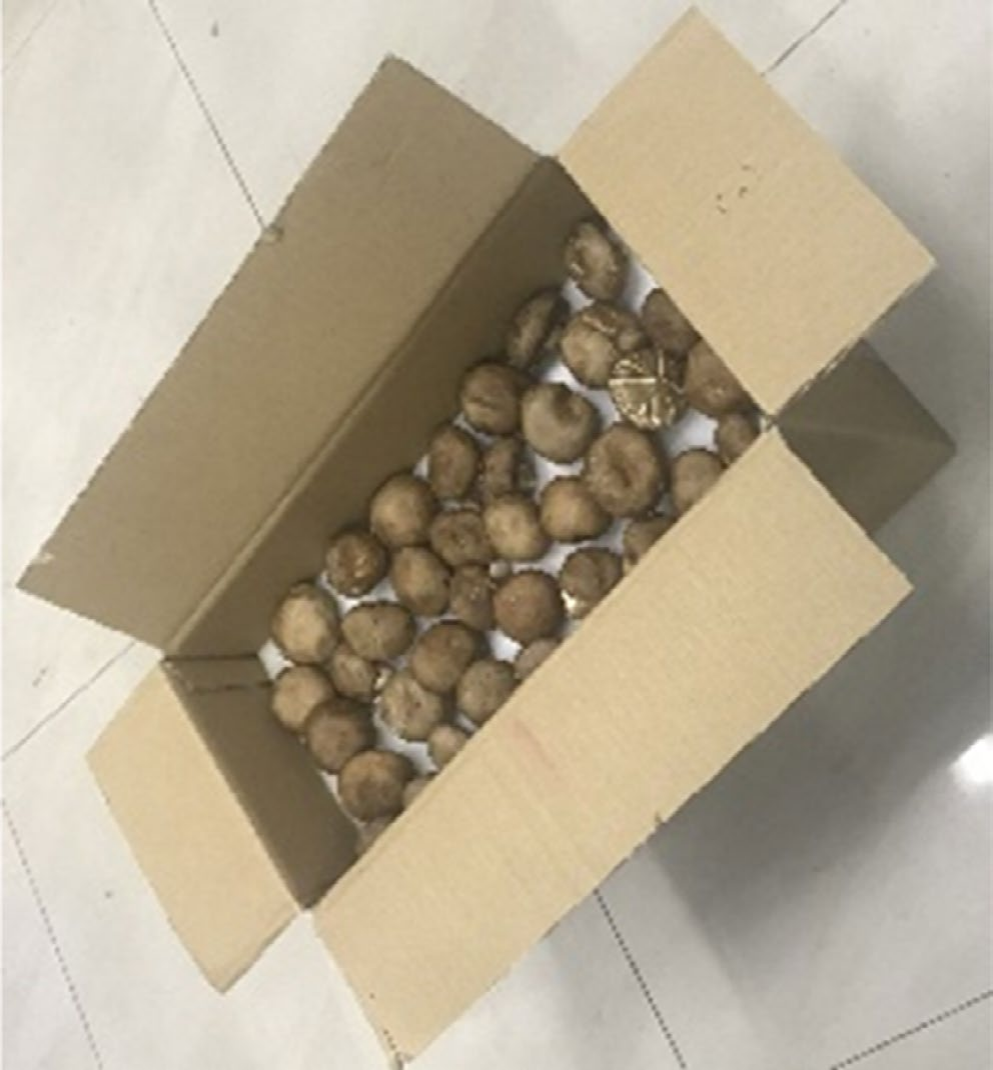
Packaging of shiitake mushrooms protected by foam cushion material

**FIGURE 2:**
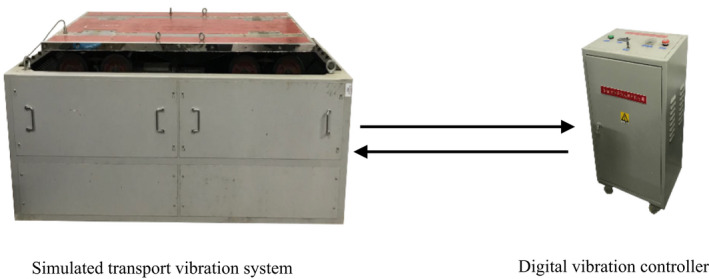
Schematic layout of vibration treatment given to shiitake mushrooms

Fresh shiitake mushrooms used in this study were purchased from a mushroom farm in Lishui, Zhejiang Province, China. Mushroom samples used in the experiment were medium sized, with weights typically ranging between 18.5 and 22.4 g. The mushrooms were spread on foam cushion material (density 16 kg/m^3^, thickness 8 mm), and five layers of the samples were placed in a package box, as shown in Figure 1. The package boxes containing mushrooms were placed on the platform of the GXYS‐200‐simulated transport vibration system (Xian Guangxin Technology Development Co., China) consisting of a digital vibration controller (Figure 2) and were subjected to vibration at 20 Hz for 4 hr. After the vibration test, the samples were transferred into polystyrene trays, wrapped with food grade PVC film, and then stored for 12 days at 5 ± 1°C. During storage, each treatment group was analyzed in triplicate after intervals of three days, for a total duration of 12 days. In this study, fresh shiitake mushrooms were separated into three groups: (1) mushrooms without vibration (controls); (2) mushrooms subjected to 4 hr of simulated transport vibration (vibrated mushrooms); and (3) mushrooms protected with foam cushion, subjected to 4 hr of simulated transport vibration (foam‐protected mushrooms).

### 2.2 Respiration rate measurement

The respiration rate was measured according to a previously reported method (Liu et al., 2016) with some modifications. Mushrooms were placed in an airtight jar containing 5 ml of NaOH solution (0.4 M). After 30 min of absorbtion, oxalic acid solution (0.2 M) was used to titrate the NaOH solution. The value was calculated based on the amount of adsorbed CO_2_.

### 2.3 Weight Loss

Weight loss was measured by estimating the weight change after an interval of three days during the storage period. The result was expressed as the percentage of weight loss with respect to the initial weight.

$$Weightloss\left(\%\right)=\frac{M_{0}‐M_{f}}{M_{0}}\times 100,$$

where M_0_ is the initial weight of the sample and M_f_ is the weight on the final day of storage.

### 2.4 Color measurement

The cap color of the mushrooms was measured with a CS‐260 colorimeter (Caipu Instrument Co. Ltd., Hangzhou, China). The values of a* (red/green), b* (yellow/blue), and L* (light/dark) were measured at three points of the cap. The browning index (BI), which indicates the extent of browning of the mushrooms (Nasiri et al., 2017), was calculated based on the following equation:

$$BI=\frac{100(x‐0.31)}{0.172},\mathrm{where}$$

$$x=\frac{a^{*}+1.75L^{*}}{5.645L^{*}+a^{*}‐3.012b^{*}}$$

### 2.5 Firmness measurement

Flesh firmness was measured with a TMS‐Pro Texture Analyzer (Food Technology Corporation, Sterling, USA) coupled to an 8‐mm cylinder probe. The cap of the mushroom was cut into cubes (35 × 35 **×** 20 mm) for the determination of flesh firmness. Penetration depth for the cube‐shaped samples was set at 4 mm. The pretest speed was 30 mm/min, the post‐test speed was 60 mm/min, and the trigger force was 0.1 N. According to the force versus time curves, the firmness value was estimated as the maximum force required to penetrate the mushroom cap.

### 2.6 Malondialdehyde (MDA) content measurement

MDA content was determined based on the method described by Liu et al., (2016) with modifications. Mushroom (10.0 g) was homogenized with 100 ml of TCA (10%). After centrifugation at 7,500 × g and 4°C for 10 min, 2 ml of the supernatant was thoroughly mixed with 2 ml of TBA solution (6 mg/ml) and subsequently heated in boiling water for 15 min. After cooling to 25 ℃, the reaction mixture was subjected to centrifugation at 7,500 × g and 4°C for 10 min. The MDA contents in mushrooms were estimated by measuring the absorbance of the supernatant at different wavelengths (450, 532, and 600 nm).

### 2.7 Statistical analysis

All values are expressed as mean ± standard deviation (*SD*). Data were subjected to one‐way analysis of variance (ANOVA) and Duncan's multiple‐range comparison test. Differences at *p* < .05 were considered statistically significant. The SPSS software package (version 13.0, SPSS Inc., Chicago, IL, USA) was used for statistical analyses.

## 3 RESULTS AND DISCUSSION

### 3.1 Respiration rates

**FIGURE 3:**
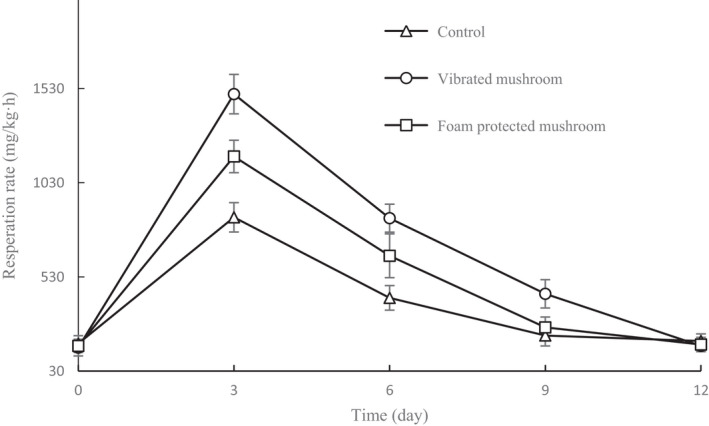
Respiration rates of shiitake mushrooms during storage for 12 d under different treatments. Data are presented as means ± *SD* of triplicates

The respiration rates of mushrooms after 12 days of storage are shown in Figure 3. In the initial stage (3 d), the respiration rate values of vibrated mushrooms, controls, and foam‐protected mushrooms were 1,500.6, 1,169.4, and 846.3 mg/kg·h, respectively. The values for all samples gradually decreased till the end of storage, but no significant difference in respiration rates was observed among the three samples after 12 d of storage (*p* ˃ .05). A similar trend was reported by Fang et al. (2016), who also focused on the respiration rate of mushrooms during storage. In the range of 3–9 d, the respiration rates of vibrated mushrooms were significantly higher than those of the controls. This was ascribed to the bruising damage from simulated vibrations that cause enhanced aerobic mitochondrial respiration due to the activation of phosphofructokinase and cytochrome oxidase (Deza‐Durand & Petersen, 2011). Respiration is a biological process in mushroom metabolism, during which fresh mushrooms consume O_2_ and release CO_2_. Respiration provides energy for anabolic reactions, and carbon skeletons for cell maintenance during storage (Fonseca et al., 2002). Because respiration rate is negatively related to the shelf life of fresh produce, many efforts to extend shelf life have focused on lowering the respiration rate (Waghmare et al., 2014). On comparing the results of vibrated and foam‐protected mushrooms, we found that the foam cushion material could lower the respiration rate. Because the respiration rate is proportional to the product deterioration rate, the foam cushion packaging during transport is useful for maintaining the quality of mushrooms during cold storage.

### 3.2 Weight loss

**FIGURE 4:**
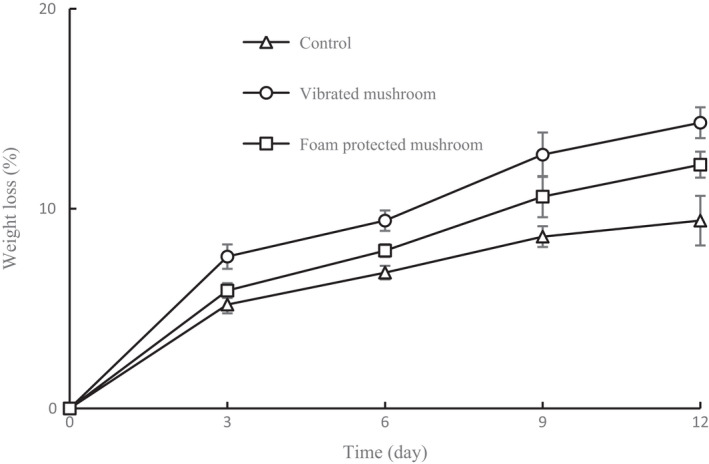
Weight loss of shiitake mushrooms during storage for 12 d under different treatments. Data are presented as means ± *SD* of triplicates

The weight loss of the samples during storage is presented in Figure 4. The results showed that the weight loss in the three samples increased as the storage period progressed. At the end of storage, vibrated mushrooms, foam‐protected mushrooms, and control exhibited weight loss of 14.3%, 12.2%, and 9.4%, respectively. The vibrated mushrooms showed more weight loss than the controls during the storage period. Furthermore, the foam‐protected mushrooms exhibited reduced weight loss compared with the vibrated mushrooms. Mushrooms are covered by a thin epidermal structure, which cannot effectively prevent rapid superficial dehydration (Jiang, 2013). Thus, weight loss in fresh mushrooms during storage is unavoidable. Weight loss greatly influences the quality and limited commercial value of fresh mushrooms. Accordingly, weight loss was a major concern for ensuring the quality of the mushrooms. Transpiration is the principal contributor to weight loss during the storage of mushrooms (Roy et al., 1995). Water loss in fruits and vegetables is governed by the moisture vapor pressure between the epidermis and the environment (Amarante et al., 2001; DíazPérez et al., 2007). Moisture transport in microstructures, particularly the cell wall and cell membrane, promotes water evaporation, leading to greater water loss (Jansasithorn et al., 2014; Wei et al., 2019). Thus, the reduced weight loss for foam‐protected mushrooms could be attributed to the minimized bruising damage on the mushroom skin, which delays the migration of moisture from the mushroom into the environment, thus suppressing transpiration intensity.

### 3.3 Color change

**FIGURE 5:**
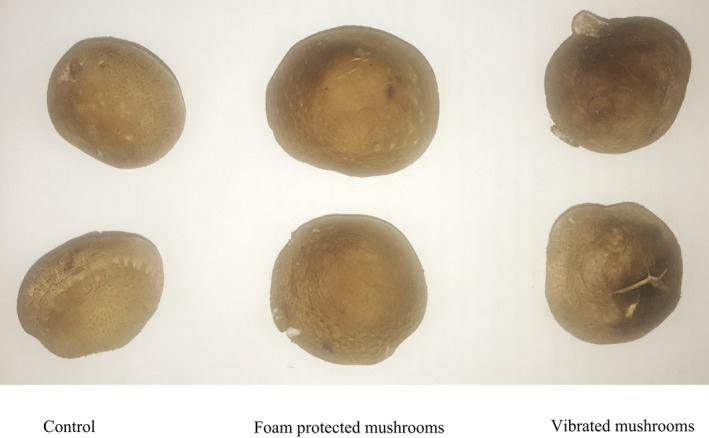
Images of shiitake mushrooms after 12 d of storage

**FIGURE 6:**
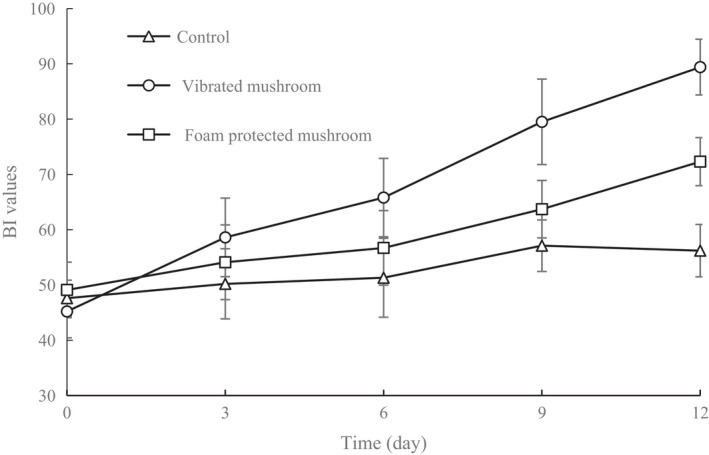
BI values of shiitake mushrooms during storage for 12 d under different treatments. Data are presented as means ± *SD* of triplicates

Images of mushroom samples at the end of storage are shown in Figure 5. Brown patches were observed on the caps of the vibrated mushrooms. In contrast, the controls and the foam‐protected mushrooms maintained a better physical appearance. Browning index (BI) is one of the main quality indicators for assessing the extent of deterioration of the surface of mushrooms. As shown in Figure 6, the BI values of mushrooms increased steadily during the storage period. At the end of storage, the BI values of the vibrated mushrooms, foam‐protected mushrooms, and the controls increased to 89.4, 72.3, and 56.2, respectively. Compared with the vibrated mushrooms, the foam‐protected mushrooms showed significantly lower BI values (*p* < .05). These results show that protection by foam material was effective at inhibiting the browning effect of mushrooms. Color is a key quality parameter, as it has a striking influence on the perception of fresh mushroom products by consumers, and affects other quality indicators, such as aroma and flavor. Postharvest browning is an economically harmful phenomenon in the mushroom industry. The discoloration of mushrooms is due to the browning reaction catalyzed by polyphenol oxidase (Jiang, 2012). This browning reaction could be triggered by the decompartmentation of tissue structure, allowing polyphenol oxidase to come into contact with polyphenols (Jolivet et al., 1998). Since protection by foam material can reduce tissue damage to mushrooms, it prevents the formation of brown patches on the surface of mushrooms, thereby improving the color and appearance.

### 3.4 Texture

**FIGURE 7:**
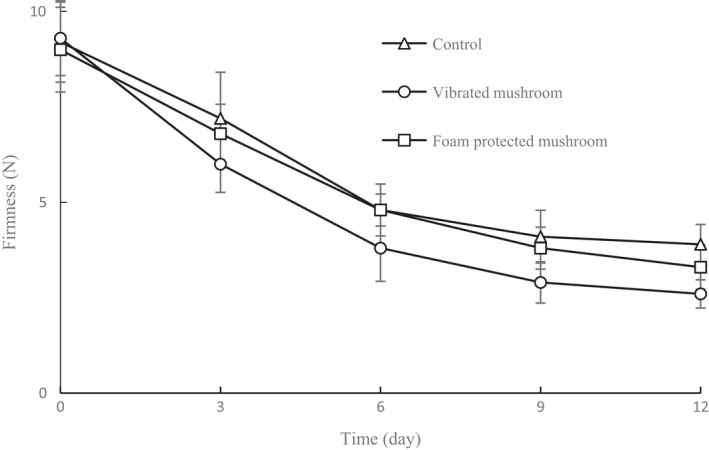
Firmness of shiitake mushrooms during storage for 12 d under different treatments. Data are presented as means ± *SD* of triplicates

As shown in Figure 7, the firmness of mushrooms gradually decreased during storage. A similar result was also reported by Akram et al. (2012). Texture serves as a crucial criterion in the overall acceptance of mushrooms and is commonly the first of many quality attributes evaluated by consumers (Zivanovic & Buescher, 2010). At the end of storage, the firmness of the vibrated mushrooms was 2.6 N, which was slightly lower than that of the control samples. This suggests that vibration has a detrimental influence on the texture of mushrooms. Compared with the vibrated mushrooms, the decline in texture of foam‐protected mushrooms was slower, indicating reduced loss of firmness. The gradual reduction in firmness of mushrooms during storage was probably due to the function of endogenous autolysins, which cause degradation of cell walls and result in softening of mushrooms (Zivanovic et al., 2000). Additionally, effects on cell membrane integrity can also lead to reduction in cell turgidity and product firmness (Beaulieu et al., 2002).

### 3.5 MDA content

**FIGURE 8:**
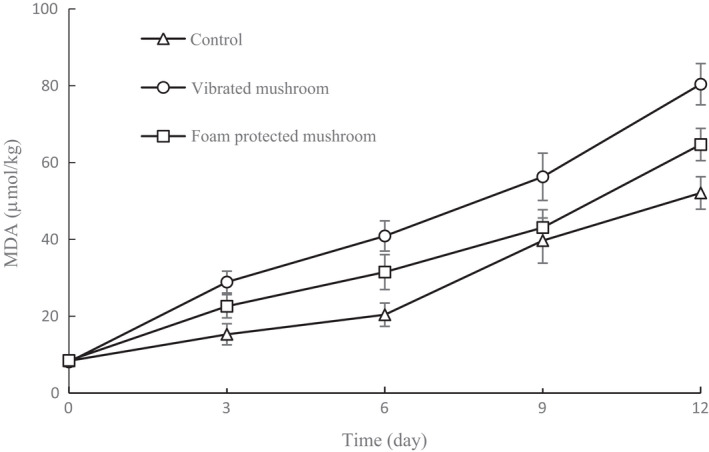
MDA contents of shiitake mushrooms during storage for 12 d under different treatments. Data are presented as means ± *SD* of triplicates

As shown in Figure 8, MDA content gradually increased in all samples over 12 d of storage. At the end of storage, the highest MDA content of mushrooms was observed in the vibrated mushrooms *(p* < .05). Foam‐protected samples showed relatively lower MDA content than the vibrated group, but higher MDA content than the control group (*p* < .05). MDA, the main reaction product of membrane lipid peroxidation, is widely used as an indicator of the degree of lipid peroxidation (Gęgotek & Skrzydlewska, 2019; Gürbüz & Heinonen, 2015). The oxidation of linolenic and linoleic fatty acids in cell membranes is catalyzed by lipoxygenase in the presence of oxygen, generating hydroperoxides that could lead to the formation of odor compounds (Myung et al., 2006). According to previous studies, mechanical vibrations may cause mechanical injury to fruits, which triggers the activation of lipoxygenase, leading to physical, biochemical, and physiological changes in fruits (Perkins et al., 2019). This suggests that foam‐protected mushrooms had lower levels of lipid oxidation during storage, probably due to the relatively integral microstructures of the cell walls and cell membranes, which caused a reduction in membrane lipid peroxidation.

## 4 CONCLUSION

The current study revealed that exposure to vibrations during road transportation is an important factor in quality deterioration of shiitake mushrooms (*Lentinus edodes*). However, shiitake mushrooms protected by foam cushioning material showed reduction in respiration rate, weight loss, extent of browning, and lipid oxidation, while preserving texture quality. Hence, the application of foam cushioning material during transportation appears to be an effective treatment to reduce the degradation of mushroom quality and extend their shelf life.

## CONFLICT OF INTEREST

The authors have declared no conflicts of interest for this article.

## Data Availability

All authors confirm that the data supporting the findings of this study are available within the article.
